# Supplementary material for: Barcode-free next-generation sequencing error validation for ultra-rare variant detection
Source: Nat Commun. 2019 Feb 28;10:977. doi: 10.1038/s41467-019-08941-4 (PMC6395625; doi:10.1038/s41467-019-08941-4)
Supplement: Supplementary file 2 — Supplementary Information [file 41467_2019_8941_MOESM2_ESM.docx]

Supplementary Information

Barcode-free next-generation sequencing (NGS) error validation for ultra-rare mutation detection

Yeom et al.

**Supplementary Notes**

**Supplementary Note 1. The possibility that PCR error leads to false variant calls during validation sequencing**

The probability of the PCR errors leading to false variant calls is extremely low. The reason is that each DNA clone is composed of many homogenous DNA molecules. Even if the PCR errors occur during early cycles of amplification, the DNA molecules with the PCR errors will be the small part of a DNA clone. Because we identified the sequences of the extracted DNA clones as the most frequently occurring sequences in each position, minor population of DNA molecules (that have PCR errors) can be filtered out. For more detailed explanation, a quantitative calculation is followed.

Before calculating a probability of having false variant calls at the validation step, we’ll assume the extreme case; more error-prone conditions. First, assume that the length of the DNA is 400 bp, the number of homogenous DNA molecules in the DNA clone is 100, and the polymerase used for PCR has a substitution error rate of 10^-4^ per base per cycle. The 400 bp length is quite a long one for the DNA molecules in NGS platform. Also, the 100 is quite a small copy number of each clones in NGS platform and 10^-4^ per base per cycle is the order of error rate of Taq polymerase, which has a high error rate than other polymerases (Phusion, KAPA, Q5, etc). Second, in the validation step, assume that the sequence of each position is determined as a sequence that occurs more than half of the whole sequences in a DNA clone. Lastly, assume that the PCR error occurs only at the first cycle of amplification in one specific position of DNA.

Probability of occurring PCR errors in the first cycle is known as followed^1^,

$P\left( k \right)=\beta\left( nl, k \right){(cx)}^{k}{(1-cx)}^{nl-k}$ (1)

where

*k* = the number of errors in the first cycle

*n* = the number of single-stranded copies before amplification

*l* = the length of DNA molecules

*c* = proportion of mismatches detected by a given method

*x* = error rate per base per cycle (error rate of polymerase)

*β* = coefficient of binomial distribution

Then, the probability of occurring one PCR error from one DNA read is

$P=\beta\left( l, 1 \right){(x)}^{1}{(1-x)}^{l-1}$ (2)

where *k* = 1, *n* = 1, and *c* = 1.

Therefore, according to our assumption, the probability of leading false variant calls due to the PCR errors in the amplification is followed.

$P(l, x, n)=\beta\left( l, 1 \right){({\frac{1}{3}(x)}^{1}{(1-x)}^{l-1})}^{0.5n}$ (3)

where *l* = 400, *x* = 10^-4^ per base per cycle, and *n* = 100.

The $\beta\left( l, 1 \right)$ is multiplied once because all 0.5*n* DNA molecules must have a same PCR error in a same position. A constant 1/3 is multiplied because three bases, except a normal base are possible candidates as the PCR error. The power of 0.5*n* means that 50 % of DNA molecules in a DNA cluster have a same PCR error. Applying the value of our assumption, the probability is about 2.4 ⅹ 10^-95^. Thus, the probability of 50 % of DNA molecules in DNA cluster having the same type of PCR error in first cycle of amplification is extremely low.

As our assumption is an extreme case, the probability will be much lower in real conditions. For example, the length of DNA (*l*) is usually shorter than 400 bp, polymerase error rate (*x*) is usually lower than 10^-4^, and the copy number of DNA molecules in a DNA clone (*n*) is larger than 100 in various NGS platforms. Also, the sequence of each position is determined as a sequence that occurs more than 70~90 % of the whole sequences in a DNA clone.

**Supplementary Note 2. Statistical analysis for the number of DNA clones to validate NGS error and PCR induced error with binomial distribution.**

We let that both of NGS error and PCR induced error (per base) be a discrete probability, which can follow binomial distribution. And we assumed that the distribution model can be normal approximation followed as below sample size formula.

$n=\frac{Z_{1-\left( \frac{\alpha}{2} \right)}^{2}\theta(1-\theta)}{d^{2}}$ (4)

where $\theta$ is unkown probability of success, d is a desired distance from the true value, $Z_{1-\frac{\alpha}{2}}$ is the unusual normal upper 100(1-$\frac{\alpha}{2}) \%$ quantile, and [a] denotes the smallest integer larger than a. With this analysis, we calculated sample size through R package, ‘binomSamSize’.

For calculating minimum sample size for identifying PCR induced error rate, about ~ $5\times{10}^{-6}$per base per doubling, we determined a confidence as 90 % and distance as $1\times{10}^{-6}$ with doublings calculated by real time qPCR such as 42 doublings or 50 doublings. Therefore, we could calculate the minimum number of bases to be analyzed as 322,021 bases.

Also, for calculating minimum sample size to verify NGS error (~0.1 %) occurred on DNA sample of known sequence, we determined a confidence as 90 % and distance as 20 % of PCR induced error. The most major factor that leads a DNA sample of a known sequence to false variant calls is PCR-induced error. Therefore, we determined the condition where PCR-induced error can be verified as the boundary condition. Because PCR-induced errors occur in lower frequencies than NGS errors, the minimum sample size required for PCR-induced error verification is larger than that of NGS error. Also, we set the distance value to 20% to verify the NGS error with a minimum number of samples that we can experiment with. We calculated the minimum sample size that can represent the whole sample, and we confirmed that when the distance value is more than 20% of the true value, the sample size in 4 repeat extractions is enough for us to handle. We could calculate the minimum number of bases to be analyzed as 145,858 bases (total 73,176 bases for validating substitution error and total 72,682 bases of indel error, respectively).

**Supplementary Figures**


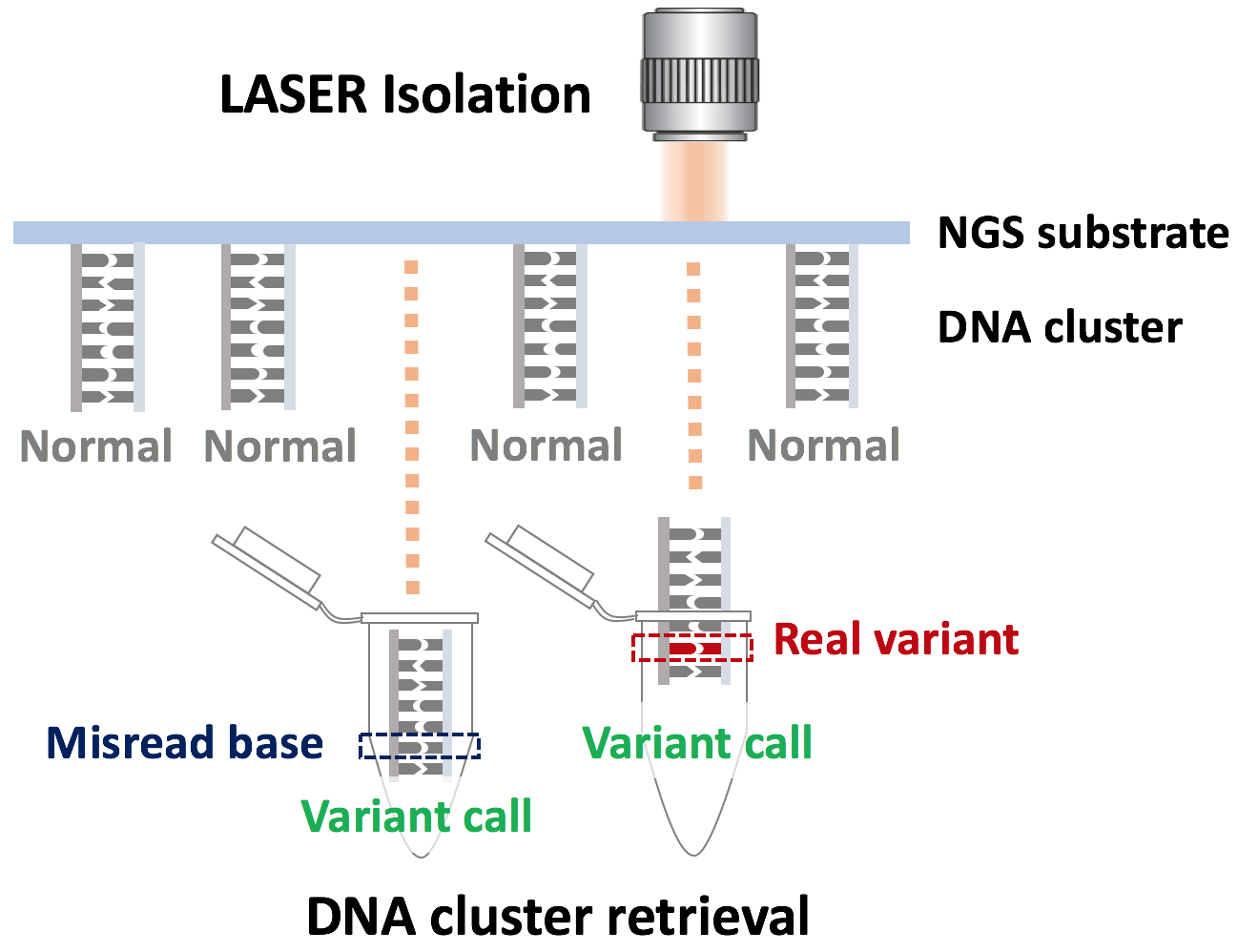


**Supplementary Figure 1.** Non-contact pulse LASER optical retrieval system.

Pulse LASER can separate DNA molecule clones of interest physically from NGS substrate, and the extracted DNA can be retrieved individually in the PCR tube. The size of the DNA molecule will not affect the performance of the physical extraction because the part where the laser is focused is the inner part of the NGS substrate. The laser ablation occurs where the laser is focused, which is set to the inner part of the NGS substrate, and the bead with the DNA clusters is located further below the surface of the substrate. This LASER retrieval system was automated to rigorously isolate target DNA clone without human intervention through in-house LabVIEW program.

**
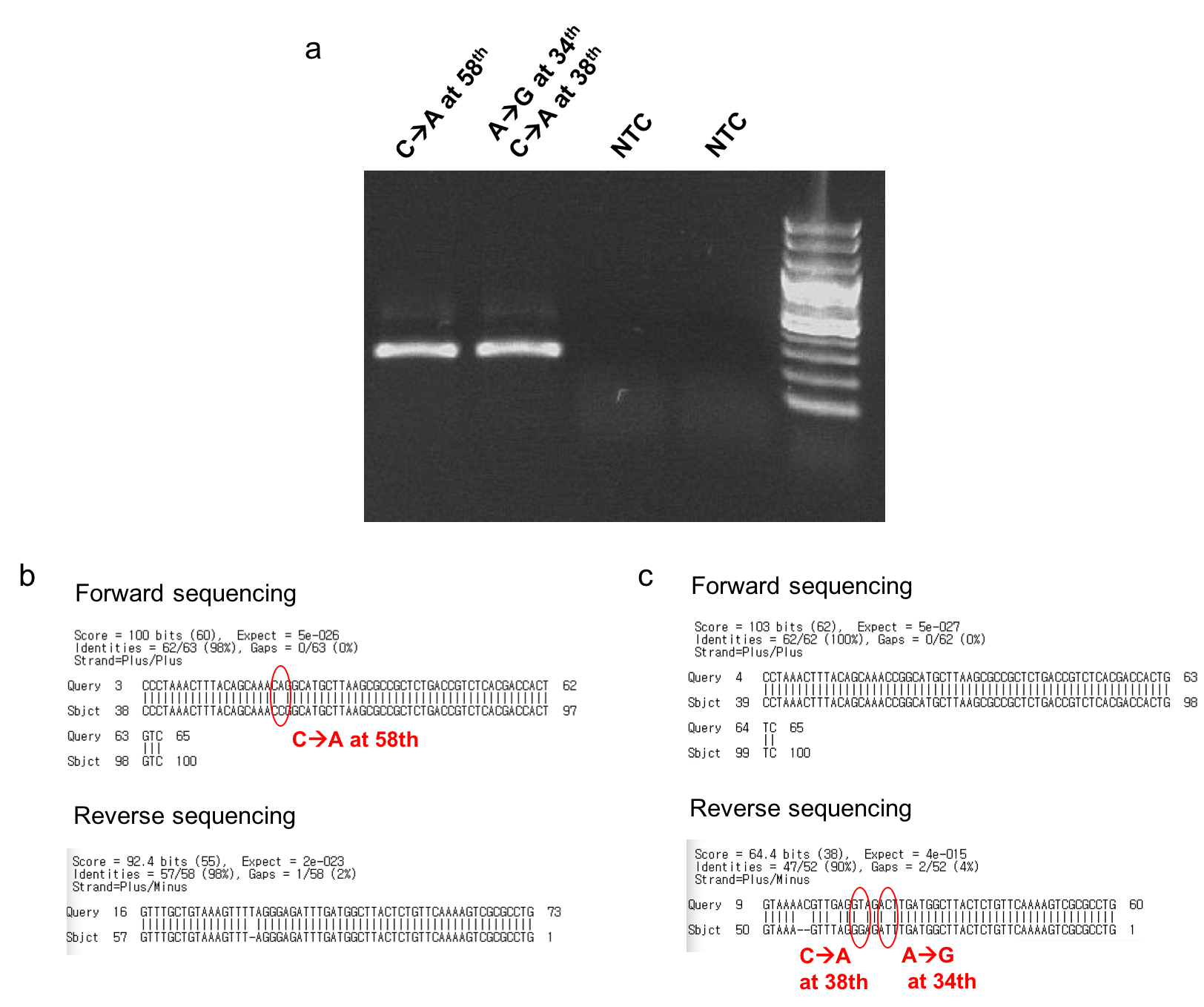
**

**Supplementary Figure 2.** Monoclonal DNA templates.

**(a)** Gel electrophoresis image of PCR product of *dapA* gene region (261 bp). Each DNA template was prepared by PCR for targeting *dapA* gene region with the *E.coli* colonies. Each PCR product was separated into the plasmid vector by Vaccinia DNA topoisomerase I and cloned (Biofact, All in One™ PCR Cloning Kit). With Sanger sequencing, we could identify each insert DNA fragments of plasmids has its own mutation at a specific position, which can be caused by polymerase error or damage such as oxidation or hydrolysis. **(b)** Sample #1 has a variant at 58^th^ position. **(c)** Sample #2 has two variants at 34^th^ and 38^th^ position. NTC = No template control; In PCR reaction, water was added instead of DNA template. BioFact™ 100 bp Plus DNA Ladder (Biofact, lane 1 and 10) included for size reference. Source data are provided as a Source Data file.


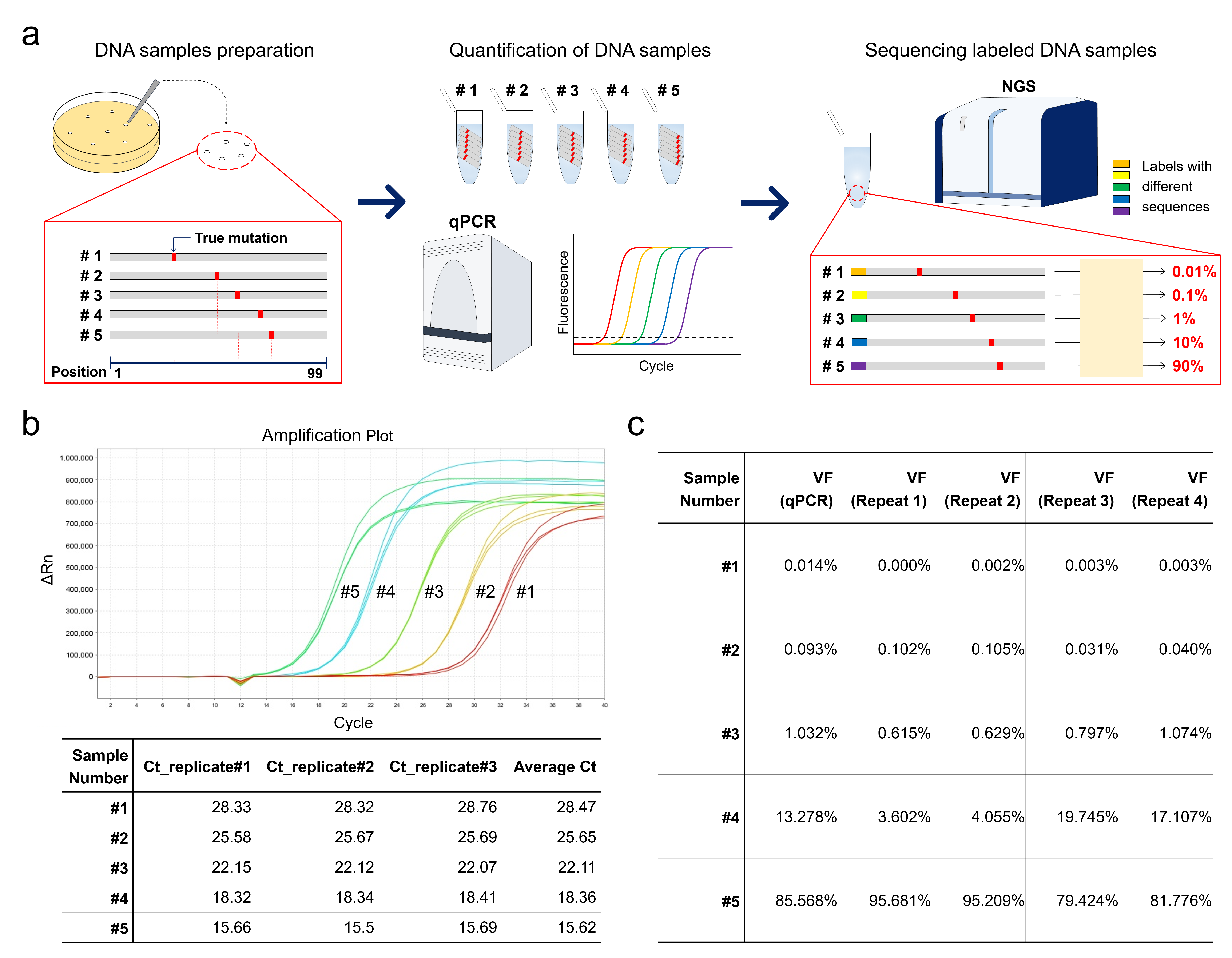


**Supplementary Figure 3.** Preparation of 5 spike-in DNA samples with variant frequency (VF) from 0.01 % to 90 %. **(a)** The whole process of preparing 5 spike-in DNA samples with different variant frequency (VF). First, colonies were picked and sequences of target region were verified by Sanger sequencing, confirming that each DNA samples had one real mutation at different positions. Secondly, DNA samples were quantified by real-time qPCR (Applied Biosystems, 7500 fast) and then diluted to make VF from 0.01 % to 90 % (0.01 %, 0.1 %, 1 %, 10 %, and 90 %). Final qPCR was done to confirm the VF of diluted DNA samples and the range of VF was from 0.002% to 95.6%. Lastly, DNA samples were labeled using different primers, followed by next-generation sequencing (Roche, 454 GS Junior). **(b)** The result of the final qPCR. Three replicates of each DNA samples were prepared and quantified by qPCR. 15 reactions (5 samples $\times$ 3 replicates) were prepared and SYBR Green I was used for the fluorescence dye. The table shows the values of the threshold cycle (Ct). With Ct values, the relative quantity of DNA samples was calculated using the relative standard curve method. **(c)** The variant frequency of each DNA samples from qPCR and four repeated experiments. VF was calculated by dividing raw data of next-generation sequencing (NGS) according to label and counting readcounts of each divided data. Source data are provided as a Source Data file.

**
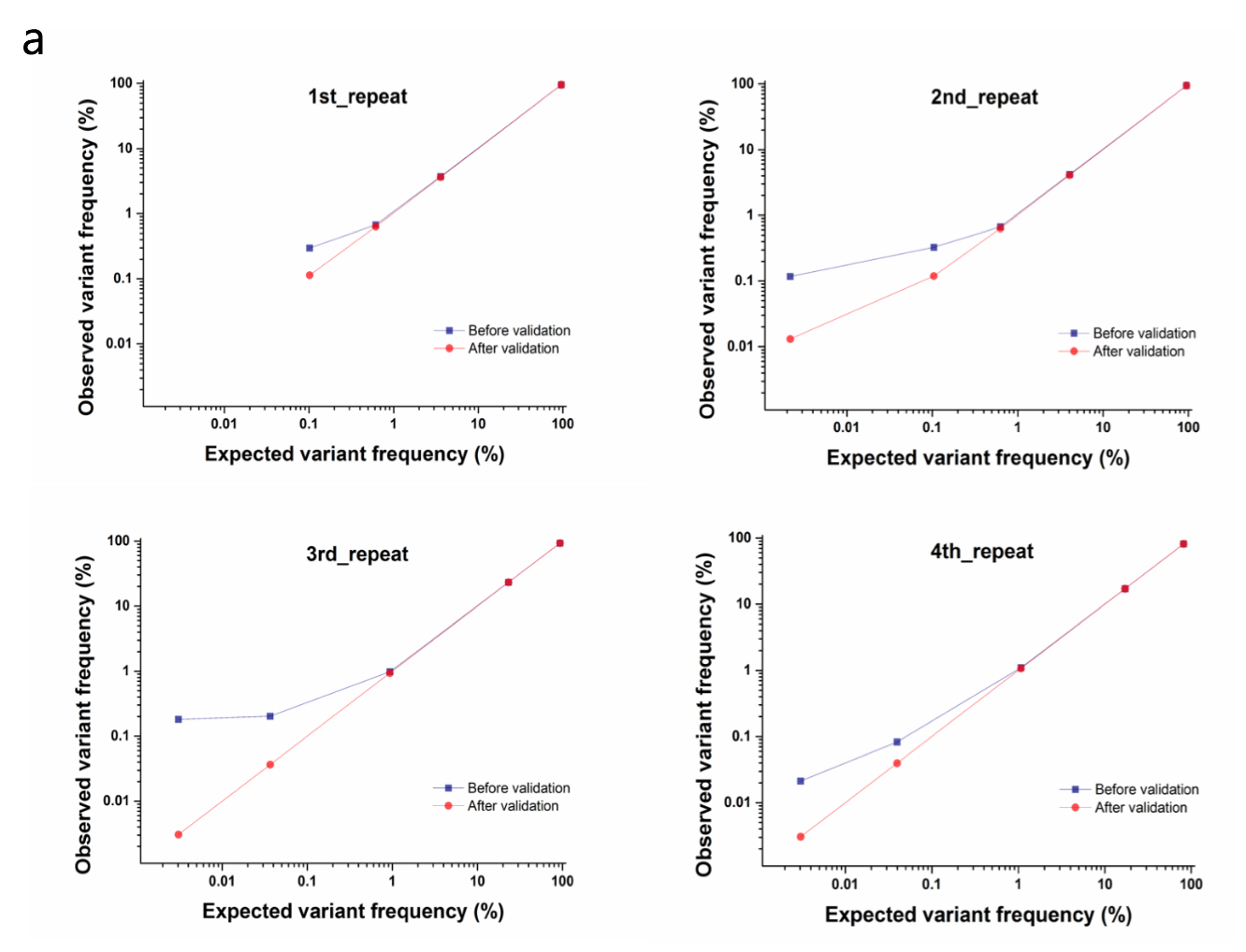
**


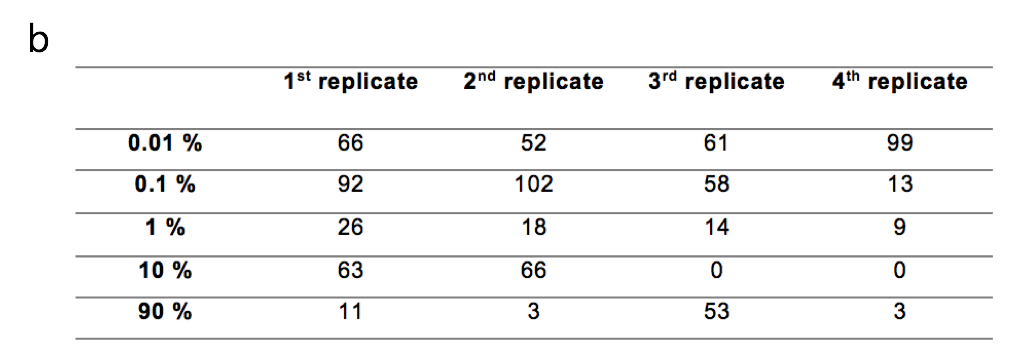

Supplementary Figure 4. Barcode-free NGS error validation for detecting spike-in DNA sample varying amounts from 95.6 % down to 0.002 % in four repeats. (a) We extracted DNA clones that have unexpected variants in raw NGS data (Supplementary Figure 3) and verified it as NGS error. Although variants (C to T at 31st position) were detected 5 times more than expected in 2nd repeat experiment, we confirmed that there were two specific variants (C to T at 31st position and C to A at 38th position) in a sequence and they could have occurred during emulsion PCR step because of two DNA templates in an emulsion. The R^2^ values can be calculated using observed variant frequency (VF) data and expected VF data. With the raw NGS result without NGS error validation, the R^2^ value was 0.77 at below VF 1 %. However, the R^2^ value was 0.98 after barcode-free NGS error validation, which means that NGS errors were well filtered. (b) The number of retrieved DNA reads for measuring the sensitivity of our validation method. We diluted DNA spike-in sample with different variant fractions of 5 orders from VF 0.01 % to 90 %, resulting in the corresponding VF measured from 0.002 % to 95.6 %. And we retrieved 806 suspicious reads from four DNA substrates (four replicate experiments). The number of reads retrieved from each replicate is 254, 240, 188, and 124 reads, respectively (see Supplementary Data 2, ID column of each sheet). Some reads have more than one variant call, so the number of suspicious variants is 819 bases. Source data are provided as a Source Data file.

**
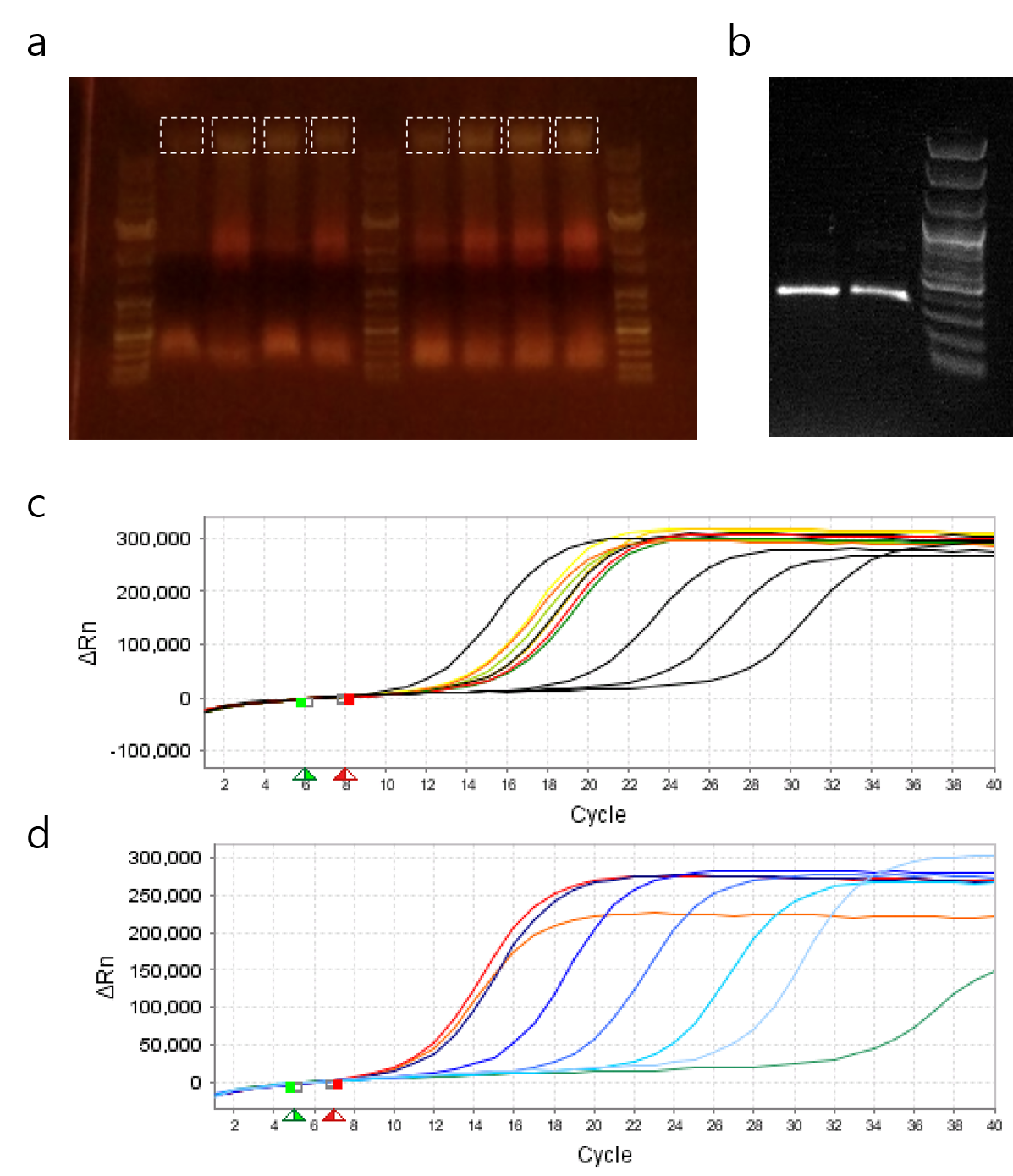
**

**Supplementary Figure 5**. PCR-induced error DNA template preparation. (a) Gel electrophoresis image of the gDNA extracted from *E.coli*. The extracted gDNA was run on a 0.5 % agarose gel, followed by purifying gDNA from the gel. (b) The purified gDNA was amplified through PCR with the primer for 1step (Supplementary Table 1). (c) Amplification plot of real-time qPCR (Applied Biosystems, 7500 fast) with the initial gDNA template before PCR amplification (520,549 copies of gDNA) (Black line: reference DNA template of ${10}^{3},{10}^{4}$,${10}^{5}, {10}^{6},\mathrm{and} {10}^{7}$copies, others: replicates of gDNA sample) (d) Amplification plot of real-time qPCR with the diluted (two times of 3/10000 and 1/100) gDNA copies after PCR (3,943,948 copies of gDNA), (Blue line: reference DNA template of ${10}^{3},{10}^{4}$,${10}^{5}, {10}^{6},\mathrm{and} {10}^{7}$copies, others: replicates of gDNA sample) Considering dilution and measured copies through qPCR, the gDNA was duplicated as 43 doublings. Source data are provided as a Source Data file.

**
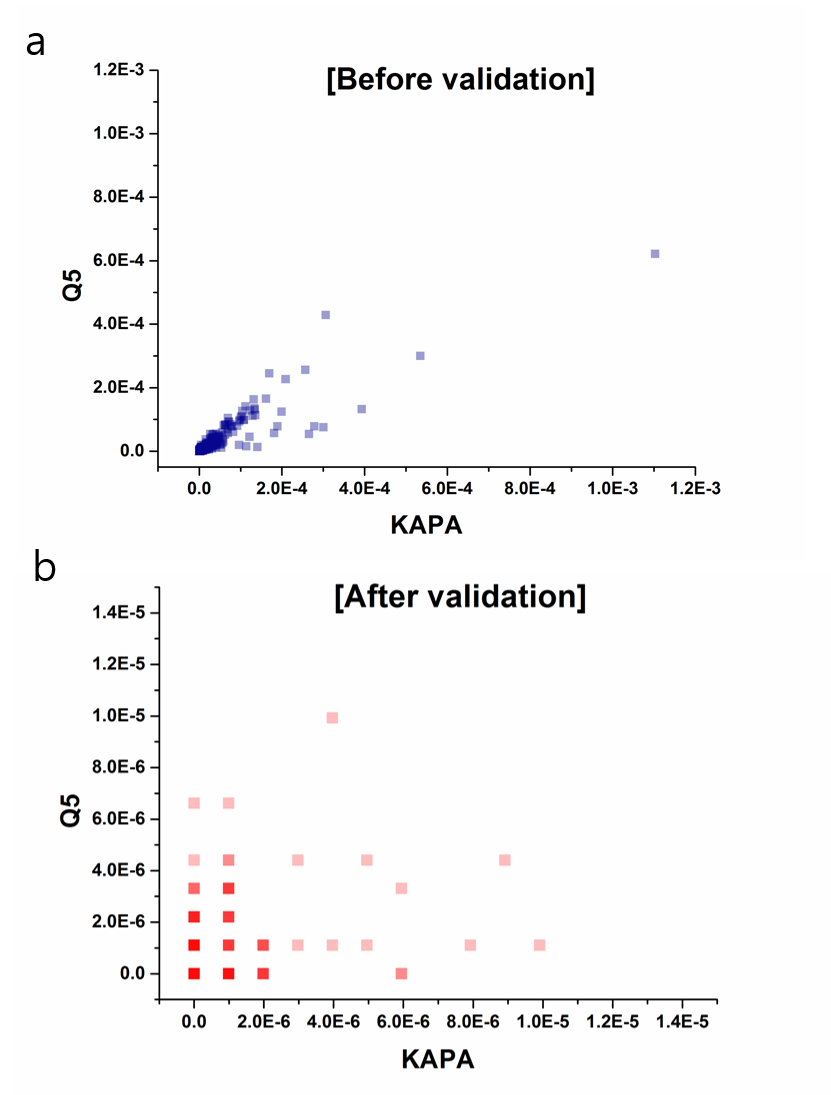
**

**Supplementary Figure 6**. Correlation analysis for DNA sample variant rates (per base) prepared by two different DNA polymerases (KAPA and Q5 DNA polymerase). From an NGS run, the characterization of NGS error (~1 % per base) should be almost identical within the same sequence, while PCR-induced error (< 0.01 % per base) will be different even more in the case of the DNA sample prepared by different enzymes. This is because the NGS error is too high to identify true variants of the lower variant frequency and reveal only the NGS error characterization by burying true variants. In this experiment, two DNA samples were prepared by Q5 polymerase (NEB) and KAPA polymerase (KAPA Biosystems) for an NGS run. And we analyzed the correlation of the variants at every sequence position (*n*=261) between the samples before and after NGS error validation. With the raw data without the NGS error validation, the error rates were similar (R^2^ = 0.88) according to same sequence. However, after NGS error validation, the error rate was not correlated to each other (R^2^ = 0.36). Source data are provided as a Source Data file.

**
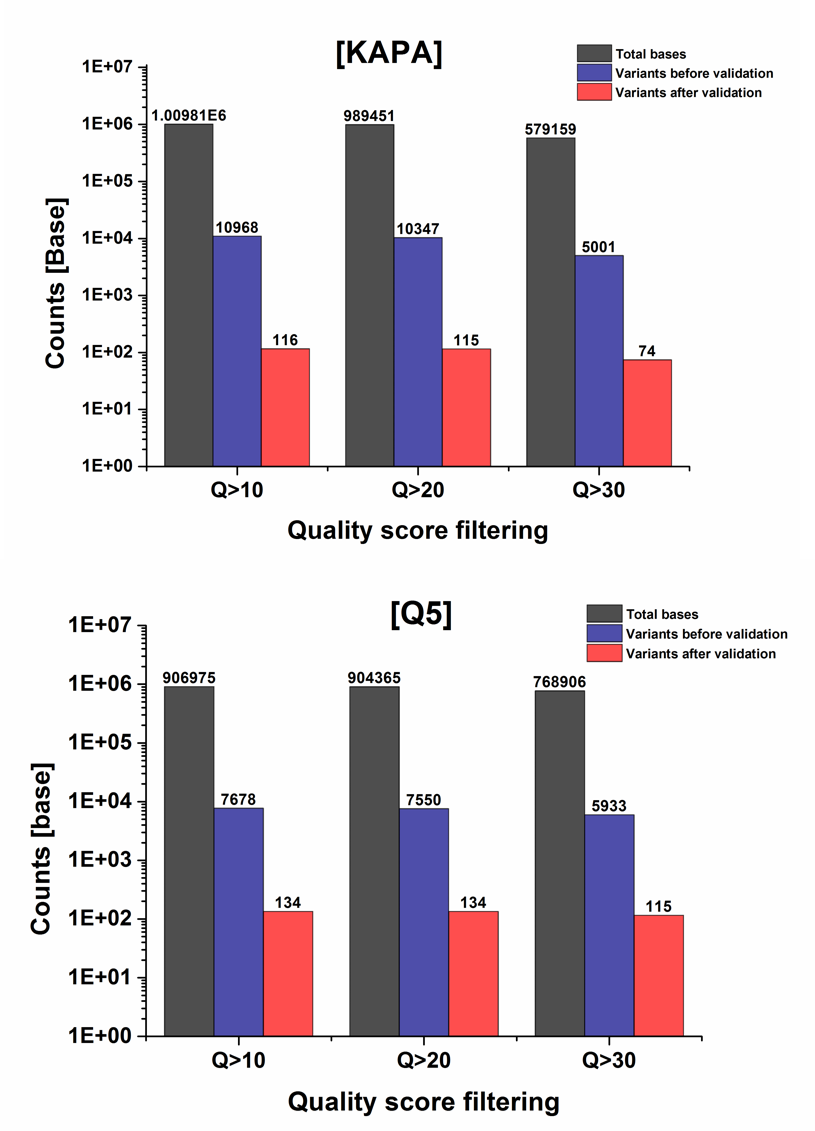
**

**Supplementary Figure 7**. Identification of true variants after trimming raw data with the Q-score threshold. Filtered variants according to Q-score threshold of the DNA template prepared by KAPA polymerase and Q5 polymerase. Source data are provided as a Source Data file.

**Supplementary Tables**

| ***dapA* gene of *E.coli*** | |
| --- | --- |
| TTACAGCAAACCGGCATGCTTAAGCGCCGCTCTGACCGTCTCACGACCACTGTCGGTGATTGGTGTCATTGGCAGGCGCAGCGTATCGGTCGCCACAAGACCCAGTTCCTTACATGCCCATTTCACCGGGATTGGATTGGGTTCGACAAATAGTTTGTTGTGTAATGGCATCAGACGCTGATTAATAACGCGTGCCTCGGCAAAATGCCCTTCTGCTGCCAGTTTGCACATCTGGGCCATATCACGCGCTGCGACGTTAGCCGTAACGGAAATAACCCCATGACCGCCCAATTGCATGAAGTCCAGCGCGCTCGCATCATCGCCGCTCAGCAGAACAAAATCATCTGAAACCAGCTCTTTGATCTGGTTTACACGCGTTAAGTTCCCTGTTGCCTCTTTGATTCCGATAATATTTTTTACTTTCGCCAGACGGCCCACCGTTTCCGGGAGCAGATCGCAGCCAGTACGGGACGGCACATTATACAGAATTTGCGGCAGGTCAGTATGCTCAGCGATGGCTTTGAAATGCTGATACAAACCTTCTTGCGACGGACGATTGTAGTAAGGGGTTACCGTCAGGCAGCCGACGATACCACTGTCATTGAAGCGCTGCGTCAGGCTAATGGCTTCCGCAGTAGCGTTAGCGCCGGTCCCGGCAATTACCGGAATGCGCCCATCAGCCAGATCCAGCGTCATCATCACCACATCAGCATGTTCGTCATGATTTAAGGTAGCGGACTCGCCAGTGGTGCCAACAGAAACGATCGCCGAAGTACCGCTGGCGACATGATAATCAATCAGTTTTTTCAAGCTAGCCCGACAGACATTACCTTTTTCATCCATCGGAGTAACAATCGCGACAATACTTCCCGTGAACAT | |
| **Monoclonal DNA template (99 bp)** | |
| Colony PCR primer (5’ 🡪 3’) | (forward) CAGGCGCGACTTTTGAAC |
|  | (reverse) ACAGTGGTCGTGAGACGGT |
| **PCR induced error accumulated DNA template (261 bp)** | |
| 1 step  (5’ 🡪 3’) | (forward) CCATCTCATCCCTGCGTGTCTCCGACTCAGhnnnnnnnnnCAGGCGCGACTTTTGAACA |
|  | (reverse) CCTATCCCCTGTGTGCCTTGGCAGTCTCAGCAGAAGGGCATTTTGCCGAG |
| 2 and 3 step  (5’ 🡪 3’) | (forward) CCATCTCATCCCTGCGTGTCTCCGACTCAG |
|  | (Reverse) CCTATCCCCTGTGTGCCTTGGCAGTCTCAG |

**Supplementary Table 1.** Gene and primer sequences

**Supplementary References**

1. Reiss, J. *et al*. The effect of replication errors on the mismatch analysis of PCR-amplified DNA. *Nucleic Acids Res.* **18**, 973-978 (1990),
