# Supplementary material for: Barcode-free next-generation sequencing error validation for ultra-rare variant detection
Source: Nat Commun. 2019 Feb 28;10:977. doi: 10.1038/s41467-019-08941-4 (PMC6395625; doi:10.1038/s41467-019-08941-4)
Supplement: Supplementary file 3 — Description of Additional Supplementary Files [file 41467_2019_8941_MOESM3_ESM.docx]

Description of Additional Supplementary Files

**File Name**: Supplementary Data 1

**Description**: This data contains the information of sequencing reads that were selected to be validated for verifying the specificity of the barcode-free next-generation sequencing (NGS) error validation method. Each data from four repeated experiments is in different sheets of this file.

**File Name**: Supplementary Data 2

**Description**: This data contains the information of sequencing reads that were selected to be validated for verifying the sensitivity of the barcode-free next-generation sequencing (NGS) error validation method. Each data from four repeated experiments is in different sheets of this file.

**File Name**: Supplementary Data 3

**Description**: This data contains the information of sequencing reads that were selected to be validated for calculating PCR-induced errors. Each data derived from three different polymerases (Phusion, KAPA, and Q5) is in different sheets of this file.
